# Exploratory machine learning analysis to characterize angioscopic features associated with atherosclerosis-related aortic dissection: an exploratory single-center angioscopic study

**DOI:** 10.3389/fcvm.2026.1784239

**Published:** 2026-05-07

**Authors:** Satoru Takahashi, Sei Komatsu, Chikao Yutani, Hiroyuki Nishi, Yoshiharu Higuchi, Nobuzo Iwa, Tomoki Ohara, Mitsuhiko Takewa, Kazuhisa Kodama

**Affiliations:** 1 Department of Cardiology, Cardiovascular Center, Osaka Gyoumeikan Hospital, Osaka, Japan; 2 Non-profit Organization Japan Vascular Imaging Research Organization, Osaka, Japan; 3 Department of Pathology, Osaka Gyoumeikan Hospital, Osaka, Japan; 4 Department of Cardiovascular Surgery, Tokai University Hachioji Hospital, Tokyo, Japan; 5 Department of Cardiology, Hyogo Prefectural Nishinomiya Hospital, Hyogo, Japan

**Keywords:** aorta, aortic dissection, machine learning, non-obstructive general angioscopy, spontaneously ruptured aortic plaques and injuries (SRAPIs)

## Abstract

**Aim:**

Aortic dissection (AD) is a life-threatening condition. Non-obstructive general angioscopy (NOGA) enables direct visualization of spontaneously ruptured aortic plaques and injuries (SRAPIs). This study evaluated the diagnostic potential and pathophysiological relevance of SRAPIs to AD.

**Methods:**

This single-center, cross-sectional observational pilot study included 56 patients with AD and 444 control patients with coronary artery disease undergoing NOGA. Healthy volunteers could not be ethically included because NOGA requires invasive catheterization. SRAPIs were classified into 13 types, and their occurrence in the aorta and bilateral common iliac arteries was analyzed using machine learning. A classification model based on Random Forest was developed, with feature selection using Least Absolute Shrinkage and Selection Operator (LASSO) regression. SHapley Additive Explanations (SHAP) and Permutation Importance were applied to interpret feature contributions.

**Results:**

Intramural blood (IB) was confirmed as the most consistent and influential SRAPI associated with AD, while puff sign (P) and salmon-pink appearance (SP) also showed strong importance. Fissure bleeding (FB) was highly frequent but showed variable importance across analytical approaches. Network analysis demonstrated preserved structural relationships after SMOTE and distinct patterns between AD and control groups.

**Conclusion:**

Aortic injury in AD may involve subintimal injury represented by IB and SP, blood or fibrin adherence to the injured vascular wall represented by P. FB may reflect a critical but non-linear pathological state rather than a purely quantitative marker. These findings reflect a selected, stable-phase cohort and require validation in larger multicenter studies.

## Introduction

Aortic dissection (AD) is a life-threatening condition caused by the disruption of the aortic wall. Traditionally, computed tomography (CT), magnetic resonance imaging, and ultrasound have been used to diagnose and assess the extent of AD (1–3). However, early diagnosis and prompt treatment remain challenging for improving AD outcomes (4). There is a growing need for an imaging modality with enhanced spatial and temporal resolution to detect early signs of acute AD (1–5). AD occurs across heterogeneous populations, and generalizable precursors remain uncertain.

Non-obstructive general angioscopy (NOGA) has been safely utilized to screen and evaluate various atherosclerosis such as spontaneously ruptured aortic plaques and injuries (SRAPIs) that may go undetected using CT (6–9). Among various types of AD, case reports have identified distinct SRAPIs in the pre-onset (10), acute (11–13), and chronic phases (14–16) of atherosclerosis-related AD using NOGA. In addition, NOGA-guided thoracic endovascular aortic repair has demonstrated favorable outcomes by enabling precise detection and coverage of reentry sites (17). However, the structural relationships among SRAPIs have not been systematically evaluated, and their role in the pathophysiology of AD remains unclear. Because direct *in vivo* validation of these angioscopic findings is difficult, analyzing relationships among SRAPI features may provide indirect insights into the underlying pathophysiology.

In medical statistics, a sufficient number of cases is required relative to the number of parameters analyzed. AD is not a rare disease; however, the detection of SRAPIs in AD using NOGA remains challenging. Sudden death is common, and emergency surgery or stent graft procedures are often necessary to save lives. In addition, invasive tests are generally avoided during conservative treatment. The limited availability of NOGA evaluating aortic atherosclerosis in patients with AD, compared to its use in patients with coronary artery disease as controls, can introduce statistical bias (18). Imbalanced data presents statistical challenges, such as class imbalance bias and overfitting (19). Medical research has increasingly adopted machine learning (ML) to address these issues. ML manages imbalanced and high-dimensional data while preserving critical variables and modelling complex interrelationships (19–21).

### Aim

This study aimed to investigate the network structure of SRAPIs identified by NOGA and to examine differences in these structural relationships between patients with and without atherosclerosis-related AD. We also aimed to better understand the underlying pathophysiology of atherosclerosis-related AD based on these structural patterns.

Machine learning was used as an exploratory analytical tool to identify key SRAPI features and to interpret their contributions within this framework, rather than to develop a clinical prediction model.

## Methods

### Study population

**Figure 1:**
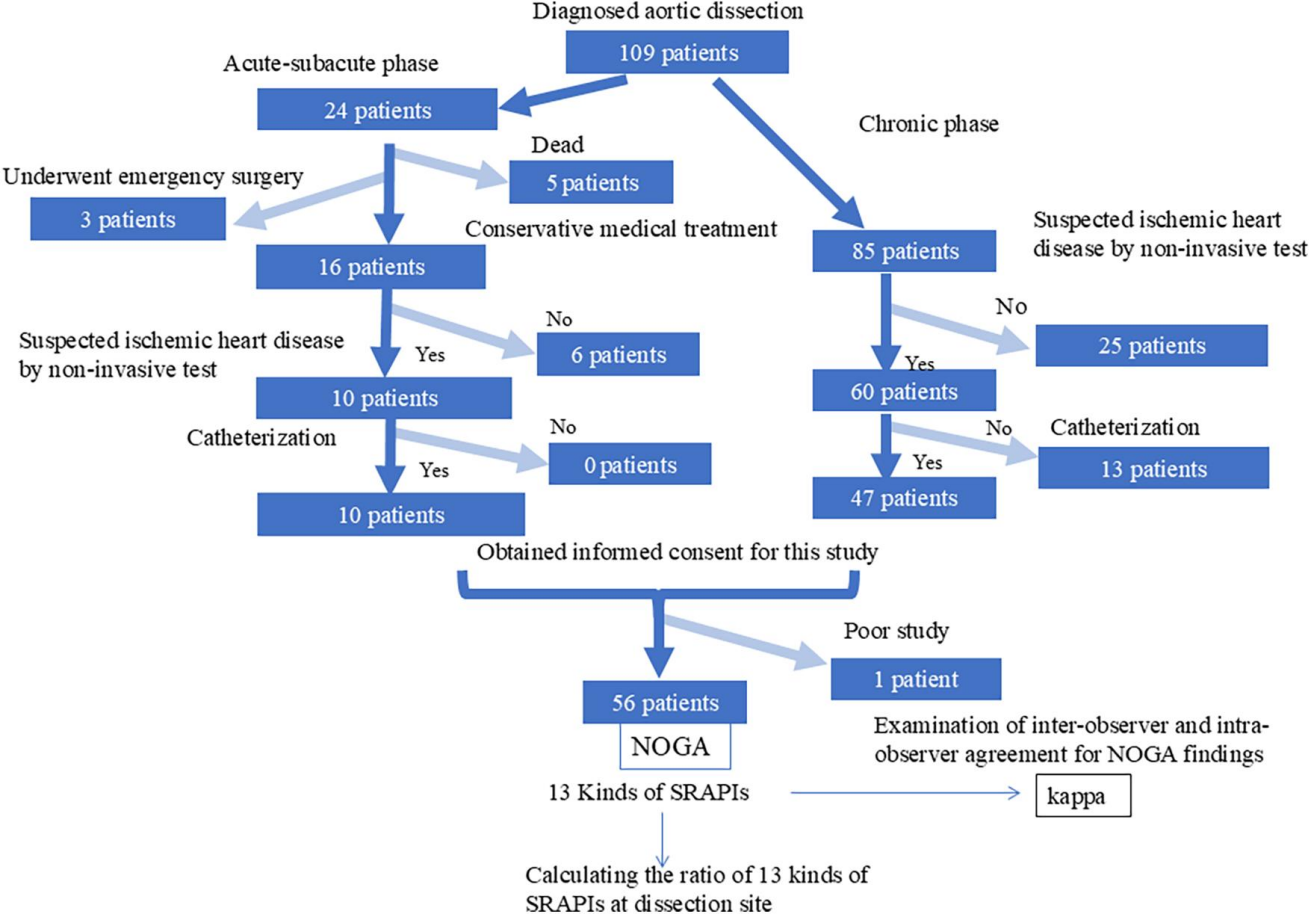
Flow diagram of the study: participant flow diagram.

This single-center, cross-sectional observational study included three patient groups at Osaka Gyoumeikan Hospital (University Hospital Medical Information Network Center ID: UMIN000053576). The first group consisted of 109 patients diagnosed with AD between November 1, 2015, and August 31, 2023 (Figure 1). Of these, 10 patients in the acute/subacute phase and 47 in the chronic phase consented to undergo catheterization and were enrolled. One patient was excluded because of structural challenges preventing NOGA observation. Accordingly, 56 cases were analyzed.

The second group consisted of 444 consecutive patients who had or were suspected to have coronary artery disease but did not have AD (Figure 1). These patients underwent cardiac catheterization with aortic evaluation for atherosclerosis besides coronary artery atherosclerosis using NOGA (6) between December 1, 2015, and September 30, 2018. The 444 patients in Group C served as the control group, allowing comparison of the prevalence and number of SRAPIs with those in the AD group. The term “Group AD” refers to the SMOTE-augmented dataset, whereas “56 patients with AD” refers to the original dataset.

**Figure 2:**
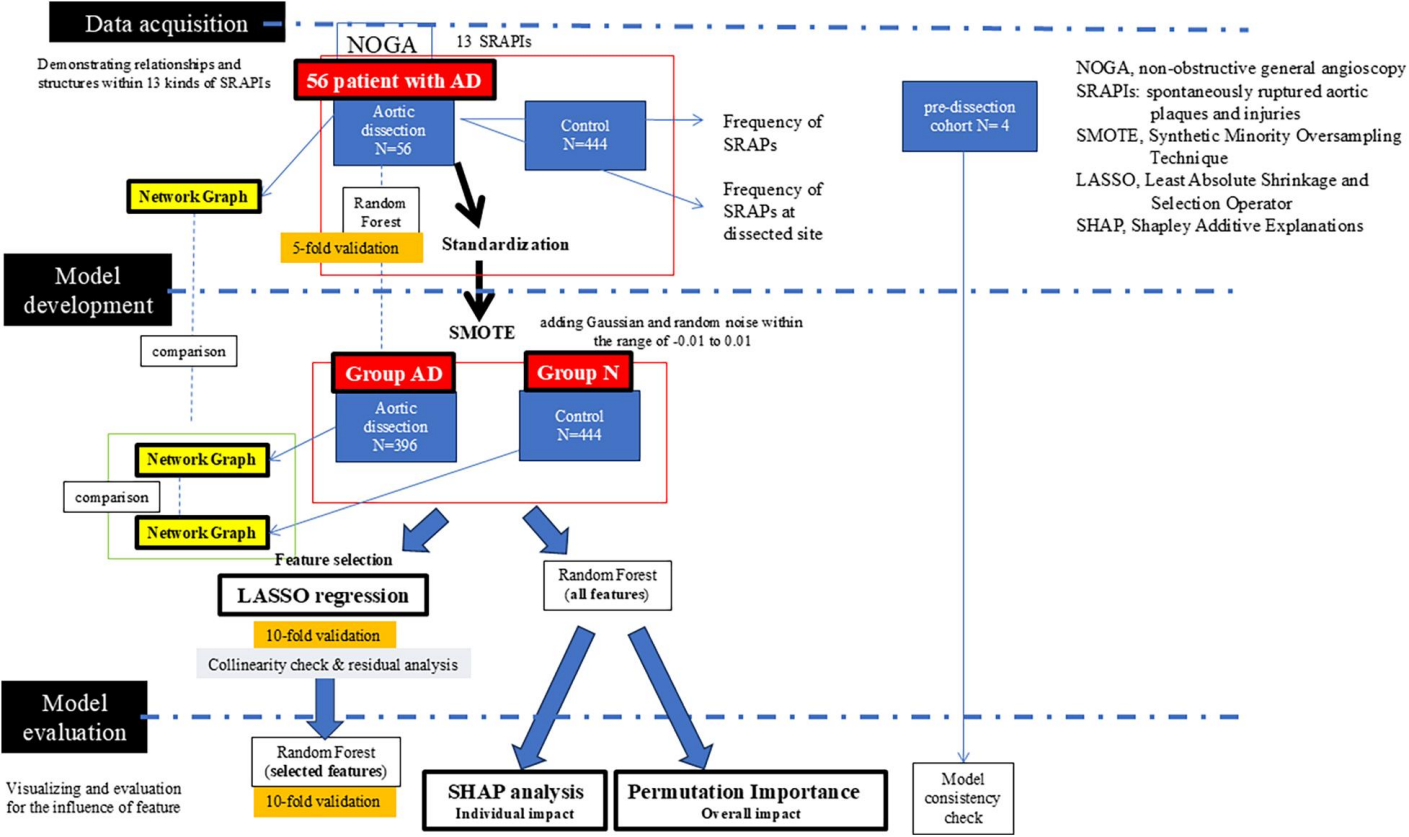
Model diagram.

We additionally identified an exploratory subcohort of four patients who had undergone NOGA during careful observation for suspected aortic atherosclerosis and were subsequently found to develop AD at the imaged segments (Figure 2). These cases were under clinical monitoring before onset, and thus were not retrospectively collected after dissection was diagnosed. This subcohort was not used for model training or validation.

Exclusion criteria included ruptured aortic dissection (AD), acute AD requiring emergency surgery, such as Stanford Type A, DeBakey Types I, II, and impending rupture. Additional exclusions were acute coronary syndrome, treatment with intra-aortic balloon pumping or percutaneous cardiopulmonary support, hemodynamic instability, contrast agent allergies, and pregnancy. This investigation was carried out following the principles outlined in the Declaration of Helsinki and approved by the Ethics Committee of Osaka Gyoumeikan Hospital (23–0021). All patients provided written informed consent before enrolment. All patients provided written informed consent before enrolment.

### Classification of aortic dissection phases

#### Diagnosis and phase classification

AD was diagnosed using non-enhanced computed tomography (CT) and CT angiography: Dual-source CT (SOMATOM Definition FLASH; Siemens, Forchheim, Germany). CT angiography findings were classified as intramural hematoma, penetrating aortic ulcer, or classic aortic dissection based on guidelines from the American College of Cardiology/American Heart Association and the European Society of Cardiology (1, 2). According to these guidelines, disease phases were categorized as follows: acute (< 2 weeks), subacute (2 weeks–3 months), and chronic (> 3 months).

#### Rationale for statistical approach and sample size considerations

Initially, we analyzed the frequency and count of 13 types of SRAPIs in both the 56 patients with AD and Group C using unique pattern analysis with the dplyr package in R. Among 444 cases in Group C, 221 unique patterns (50%) were identified for frequency and 351 unique patterns (81%) for the count of SRAPIs. In contrast, among the 56 patients with AD, 44 unique patterns (79%) were identified for frequency, and 56 unique patterns (100%) for the count of SRAPIs. These findings indicate that SRAPIs exhibited strong diversity. Due to the large number of unique patterns, the dataset became sparse. In addition, the number of AD cases was small relative to the 13 SRAPI parameters. A substantial difference was observed in the distribution of unique patterns across the groups. Therefore, conventional linear multivariate analysis alone was deemed insufficient. Instead, we applied the Least Absolute Shrinkage and Selection Operator (LASSO) regression to address data sparsity rather than relying on traditional multivariate analyses or propensity score matching. In addition, multiple nonlinear regression methods were used for comparison.

The control group size was set at approximately 4–10 times that of the case group to ensure statistical robustness (22). Previous studies suggest that this ratio enhances the reliability of rare event detection. Four types of SRAPIs were present at the AD site in fewer than 5% of cases, necessitating a sufficiently large control group to identify distribution differences in these rare findings. This approach may improve detection power and reduce statistical errors.

### Data preparation

We defined two datasets: (1) the original dataset (56 patients with AD and 444 controls; pre-SMOTE), and (2) the SMOTE-augmented dataset (396 AD and 444 controls; post-SMOTE). To address the class imbalance (1:8 ratio), the Synthetic Minority Oversampling Technique (SMOTE) (23) was applied, increasing the number of AD cases to 396. Gaussian and random noise (± 0.01) were added to the synthetic samples to prevent overfitting. The performances of both models were evaluated, with Random Forest classifiers trained using the Caret package in R. We assessed model performance using accuracy, sensitivity, specificity, and the kappa coefficient on both pre-SMOTE and post-SMOTE balanced datasets with noise augmentation.

After confirming the resolution of chest and/or back pain, normalization of blood pressure through diet, optimal medical therapy, and absence of aortic rupture symptoms on CT, The NOGA system utilized either a VISIBLE Fiber (FT-203F, Fiber Tech Co. Ltd., Tokyo, Japan) or a smart eye fiber (SURGTEC Co. Ltd., Tokyo, Japan), with a default console (Inter-tec Medicals Co. Ltd., Osaka, Japan). NOGA was performed using the dual infusion method (6) NOGA was used to assess aortic wall injuries from the ascending aorta to the ipsilateral common iliac artery, including the dissection site. In all patients, aortic atherosclerosis was evaluated through NOGA observations following coronary angiography or percutaneous coronary intervention (6, 8)

#### The definition of SRAPIs

Thirteen types of SRAPIs, listed in (Supplementary Table S1), were analyzed. SRAPIs were defined according to the consensus standards (24) To determine the SRAPIs, all images were reviewed by two independent investigators blinded to patient identity. The number and prevalence of SRAPIs were recorded and standardized.

### Intra- and inter-observer variability

Two physicians, each with >10 years of experience in NOGA, reviewed the observations and excluded duplicates. To assess intra-observer variability, a single examiner evaluated 20 previously unobserved SRAPI images using NOGA, with agreement quantified using kappa statistics. For inter-observer variability, the two independent examiners with similar experience reviewed a separate set of 20 previously unobserved SRAPI images using NOGA, and kappa statistics were similarly used to evaluate agreement.

### Data analysis

The analysis consisted of three components: 1 descriptive analysis (pre-SMOTE), 2 network analysis, and 3 machine learning analysis (post-SMOTE).

### Descriptive analysis

Analyses were primarily performed using the original dataset (pre-SMOTE), unless otherwise specified.

Comparison of Demographic and Clinical Characteristics between Group AD and Group C: Patient demographic and clinical characteristics were analyzed. The chi-square test was used to compare sex between the AD and C groups. The Mann–Whitney *U*-test was applied to compare age, heart rate, systolic and diastolic blood pressure, C-reactive protein, hemoglobin A1c, fasting blood sugar, total cholesterol, triglycerides, high-density lipoprotein cholesterol, and low-density lipoprotein cholesterol levels between the two groups.

The Prevalence of SRAPIs in the Dissected Sites: The prevalence of the 13 SRAPI types at the dissected sites was calculated among 56 patients with AD.

Comparison of SRAPI Distribution and Association between Groups AD and C (pre-SMOTE dataset): The distribution of SRAPIs between Groups AD and C was compared using the chi-square test.

The Number of SRAPIs in Groups AD (post-SMOTE dataset) and C (original dataset): Subsequently, analyses of SRAPI counts were performed using the SMOTE-augmented dataset. The number of 13 types of SRAPIs in the aorta and common iliac artery was calculated for the Groups AD and C.

Exploratory observations were performed in a separate subcohort of four patients.

The number of SRAPIs was counted in patients who had undergone NOGA during careful observation for suspected aortic atherosclerosis and were subsequently found to develop AD.

This analysis aimed to examine the structural relationships among SRAPI features and to assess whether key SRAPIs identified by the model were present before AD onset.

This analysis also served as a preliminary, hypothesis-generating step to support network-based analysis (25, 26) because direct validation is difficult in AD patients.

Network Analysis (structural relationships) (Figure 2).

Network graphs (27) were created to analyse the relationships among SRAPIs in the 56 patients with AD, Group AD, and Group C. Analyses were performed using both pre-SMOTE and post-SMOTE datasets for comparison. Each SRAPI type was defined as a node, and pairwise Pearson correlation coefficients were calculated based on the presence of each SRAPI feature. Undirected weighted networks were constructed, with edges defined between nodes with an absolute correlation coefficient greater than 0.3, and edge weights set to the corresponding correlation values. Network properties were quantified using density, diameter, global clustering coefficient (transitivity), and degree centrality. Network similarity between groups was assessed using multiple complementary approaches. First, the Jaccard similarity index was calculated based on binary edge sets derived from thresholded correlation networks (|r| > 0.3). Second, weighted Jaccard similarity was computed to account for edge weights, defined as the ratio of the sum of minimum edge weights to the sum of maximum edge weights across networks. This was used to reflect differences in edge weights. Third, overall network similarity was evaluated by computing the Pearson correlation between the upper triangular elements of the correlation matrices. These complementary metrics were used to capture both binary edge overlap and weighted structural similarity. Comparisons were performed between the 56 patients with AD (pre-SMOTE) and Group AD (post-SMOTE), and between Groups AD and C.

### Machine learning analysis (post-SMOTE)

Feature Selection (Figure 2): To predict group membership (Group AD vs. Group C), we utilized logistic regression with LASSO regularization, which aids in variable selection by shrinking less informative predictors to zero, reducing the risk of overfitting (28). A 10-fold cross-validation approach was applied to optimize the regularization parameter, where the dataset was split into 10 parts, with nine used for training and one for testing, and the process repeated 10 times to ensure each subset served as the test set. Performance metrics, including area under the curve, accuracy, sensitivity, and specificity, were computed for each fold and averaged across all folds. Residual and multicollinearity analyses were conducted. Other machine-learning algorithms, such as support vector machines and gradient boosting, were not included because of the limited sample size and concerns about overfitting. In addition, since the interpretation of data from 13 types of SRAPIs was required, a linear SVM was not adopted.

Feature Importance Analysis for Individual Impact: Feature contributions were evaluated using SHapley Additive exPlanations (SHAP) analysis (29) via the shap package in Python, assessing the impact of individual SRAPIs on classification outcomes. Other MLs were not included due to the limited sample size, concerns about overfitting, and the need for interpretability of SRAPIs A Random Forest model, implemented with RandomForestClassifier from the scikit-learn library, was used for classification. The dataset was divided into training (80%) and testing (20%) sets using train_test_split. The model was trained with RandomForestClassifier. A feature importance plot was generated based on the SHAP values to visualize each feature's contribution to the model's predictive performance, highlighting the direction of influence (positive or negative) and variability in effect.

Feature Importance Analysis Using Permutation Importance for Overall Impact: After training the Random Forest model, its performance was evaluated by predicting the test data, and classification accuracy was computed using accuracy_score. Permutation Importance was assessed using the permutation_importance function from scikit-learn (30). Each feature's values were randomly shuffled 10 times, and the model's accuracy was measured after each shuffle to assess the relative importance of each feature. The results were reported as the mean importance (importances_mean) and standard error (importances_std) for each feature. A bar plot was generated using Matplotlib, with standard errors represented as error bars to visualize feature importance.

### Statistical analysis

SHAP and Permutation Importance analyses were conducted using Python (version 3.10.0rc1; Python Software Foundation, Delaware, USA). All other statistical analyses were performed using the open-source statistical software R (version 4.4.2; The R Foundation for Statistical Computing, Vienna, Austria).

## Results

### General characteristics and data reliability assessment

Comparison of Demographic and Clinical Characteristics between Group AD and Group C: There were no missing data for the variables analyzed. None of the patients had inherited diseases or autoimmune disorders, such as Marfan syndrome or aortitis, and none had malignancies before or 3 months after catheterization (Table 1). presents the patient characteristics. All continuous variables were analyzed without arbitrary categorization. Systolic and diastolic blood pressure, as well as C-reactive protein levels were significantly higher in Group AD than those in Group C (*P* < 0.001, *P* = 0.012, and *P* = 0.001, respectively). Furthermore, the proportion of male patients was significantly higher in Group AD than in Group C.

**Table 1:** The comparison of patients’ characteristics between group AD and group C.

Variable	Group AD	Group C	*p*-value
Age	75.0 [68.0, 80.0]	73.0 [66.0, 79.0]	0.261
Gender male (*N*, %)#	45,80	283, 63	0.014
Heart rate	69.5 [61.75, 77.25]	71.0 [64.0, 79.0]	0.251
Systolic blood pressure (mmHg)	147.5 [126.8, 163.5]	132.0 [120.0, 145.0]	<0.001
Diastolic blood pressure (mmHg)	78.0 [67.0, 85.25]	70.0 [62.0, 80.0]	0.012
C-reactive protein (mg/dl)	0.23 [0.10, 0.45]	0.13 [0.08, 0.23]	0.001
Hemoglobin A1c (%)	5.8 [5.5, 6.3]	5.9 [5.6, 6.5]	0.145
Fasting blood glucose	103.0 [93.0, 118.25]	108.5 [93.0, 137.0]	0.191
Total cholesterol (mg/dl)	181.0 [152.0, 195.0]	174.0 [150.0, 204.0]	0.907
Triglyceride (mg/dl)	120.5 [81.5–197.8]	120.0 [87.0, 174.0]	0.928
High-density lipoprotein cholesterol (mg/dl)	46.0 [37.75, 56.25]	48.0 [41.0, 57.0]	0.359
Low-density lipoprotein cholesterol (mg/dl)	92.0 [78.5, 116.0]	95.0 [73.0, 120.0]	0.895

Data are expressed as median [interquartile range] or number, %. Mann–Whitney test #: Chi-square test.

Types and Segments of Dissection Sites Evaluated Using CT Angiography: Among the 56 patients with AD, there were 46 cases of aortic intramural hematoma, 9 cases of penetrating aortic ulcer, and 11 cases of patent false lumen. Some patients exhibited more than one of these aortic pathologies. In addition, concomitant aortic aneurysms were observed in four patients. The infrarenal aorta was affected in 35 cases, the aortic arch in 19 cases, and the descending thoracic aorta in 16 cases. Dissections involving the common iliac arteries were observed in 11 cases, while the suprarenal abdominal aorta was affected in 4 cases. In 14 patients, the dissection involved two or more aortic segments. Eight patients had dissections spanning from the aortic arch to the descending thoracic aorta, while 6 had dissections extending from the suprarenal to the infrarenal abdominal aorta.

Angioscopic Finding of SRAPIs: Representative images are depicted in (Supplementary Figure S1), and a corresponding video in AD is provided in Video 1. Intra-observer and inter-observer agreements of diagnosing SRAPI images were both substantial, with Cohen's kappa and weighted kappa values of 0.80 (95% CI: 0.53–1.00) and 0.80 (95% CI: 0.53–1.00), respectively.

### Descriptive analysis

**Figure 3:**
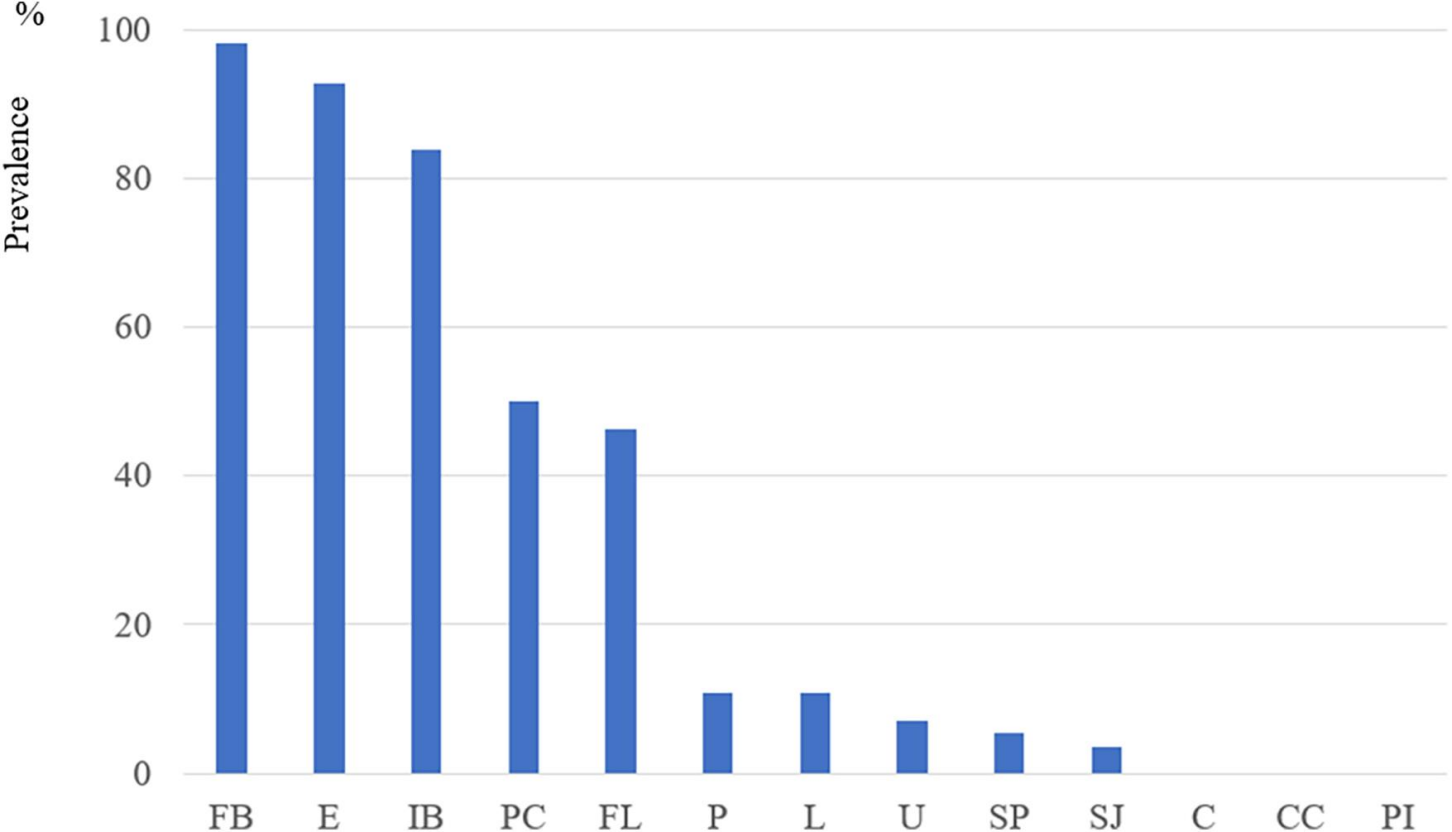
Prevalence (pre-SMOTE) and number (post-SMOTE) of SRAPIs in aortic dissection (AD) and control **(C)** groups. **(A)** Prevalence of SRAPIs at dissected sites in 56 patients with AD (pre-SMOTE, original dataset) Fissure bleeding (FB), erosion **(E)**, and intramural blood (IB) were the most frequently observed features, followed by puff-chandelier rupture (PC) and flap (FL). Other features, such as puff sign **(P)**, angioscopic ulcer **(U)**, and strawberry-jam appearance (SJ), had lower prevalence.

The Prevalence of SRAPIs in the Dissected Sites: The prevalence of SRAPIs at the dissected sites was as follows: FB, 98.2%; E, 92.9%; intramural blood (IB), 83.9%; PC, 50%; FL, 46.4%; puff sign (P) and Loft appearance (L), 10.7% each; angioscopic ulcer (U), 7.1%; salmon-pink appearance (SP) 5.4%; and strawberry-jam appearance (SJ), 3.0%. No cases of chandeliers, cotton-candy appearance (CC), or peeled intima (PI) were observed (0%) (Figure 3).

Comparison of SRAPI Distribution and Association between Groups AD and C (pre-SMOTE dataset): The prevalence of intramural blood (IB) was significantly higher in Group AD than in Group C across the 13 SRAPI types. Fissure bleeding (FB), erosion (E), puff (P), puff-chandelier rupture (PC), and flap (FL) were also frequently observed in patients with AD (Supplementary Table S2).

**Figure 4:**
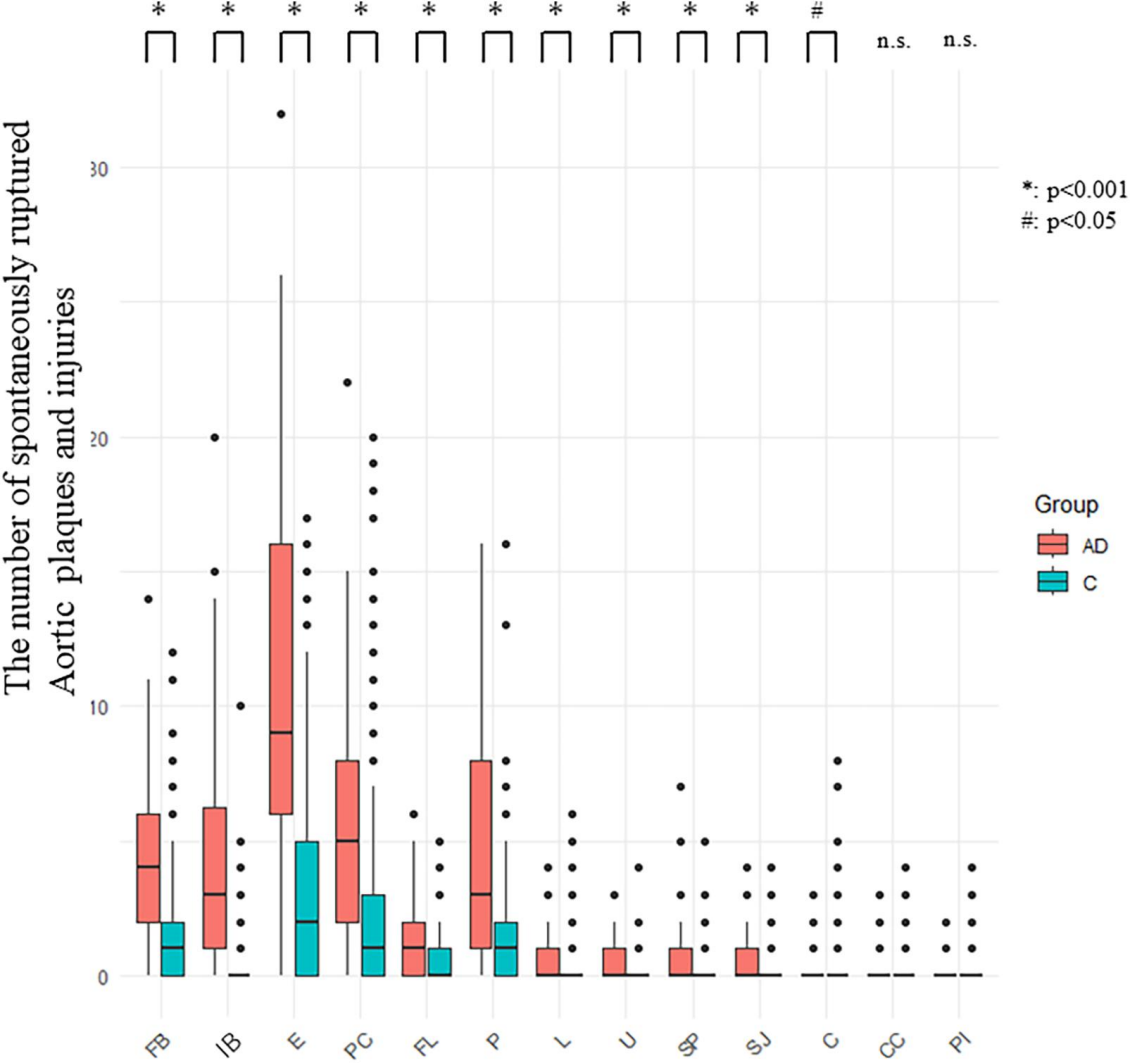
Number of SRAPIs in the aorta and common iliac artery in groups AD (post-SMOTE, SMOTE-augmented dataset) and C (original dataset) SRAPIs were generally more abundant in group AD than in group C. Intramural blood (IB), erosion **(E)**, puff-chandelier rupture (PC), and fissure bleeding (FB) showed greater variation. Box plots illustrate medians and interquartile ranges. SRAPIs, Spontaneously ruptured aortic plaques and injuries; AD: Aortic dissection.

The Number of SRAPIs in Groups AD (post-SMOTE dataset) and C (original dataset): Figure 4 illustrates the distribution of the 13 types of SRAPIs in the aorta and common iliac artery in the 56 patients with AD and Group C. The numbers of FB, E, PC, FL, P, L, U, SP, SJ, and IB in Group AD were higher than in Group C. The number of C was lower in Group AD. (*p* < 0.05) There was no difference in the number of CC and PI between Group AD and C (Figure 4).

Observations in the Pre-dissection Cohort: In the exploratory pre-dissection cohort, FB and IB were detected at the future dissection sites in all patients. 2 patients were detected SP (Supplementary Table S3).

**Figure 5:**
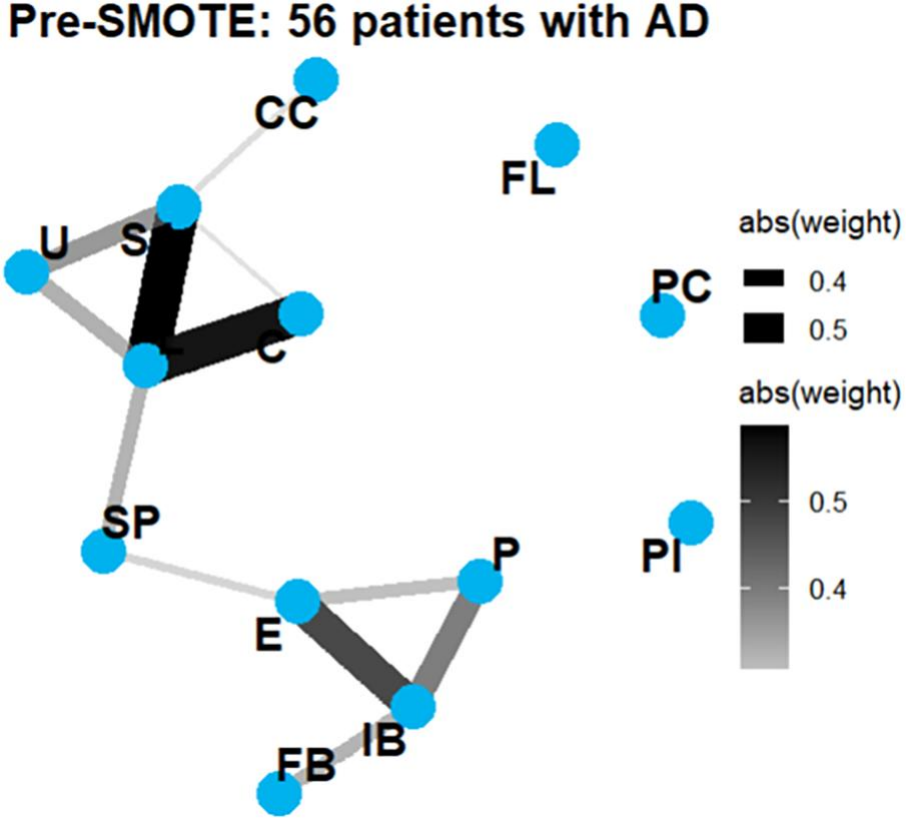
Network graphs in 56 patients with AD (Pre-SMOTE).

**Figure 6:**
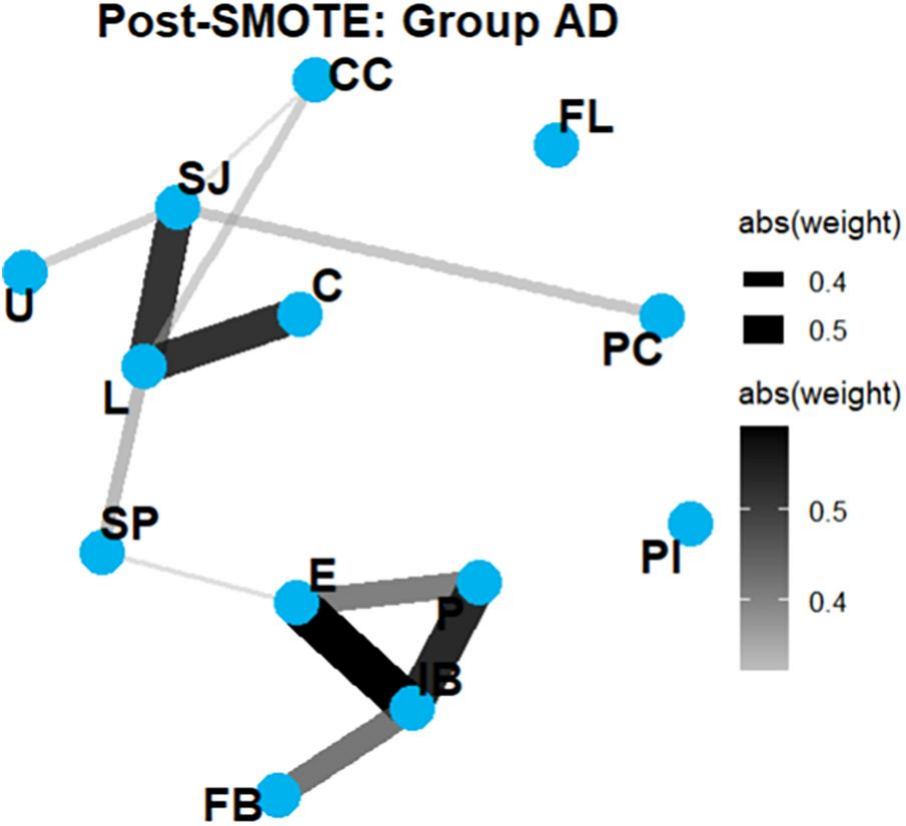
Network graphs in group AD (post-SMOTE). Quantitative analysis demonstrated high structural similarity between the pre- and post-SMOTE AD networks (Jaccard index: 0.714; weighted Jaccard: 0.686; correlation similarity: r = 0.964), supporting preservation of network topology after oversampling.

Network Graph Comparison: 56 patients with AD (Pre-SMOTE) vs. Group AD (Post-SMOTE) (Figures 5, 6).

The network diameter remained unchanged, and the global clustering coefficient remained nearly constant. The network structure was well preserved after SMOTE, with high similarity between the pre-SMOTE and post-SMOTE networks (Jaccard index: 0.714; weighted Jaccard: 0.686; correlation matrix similarity: r = 0.964).

**Figure 7:**
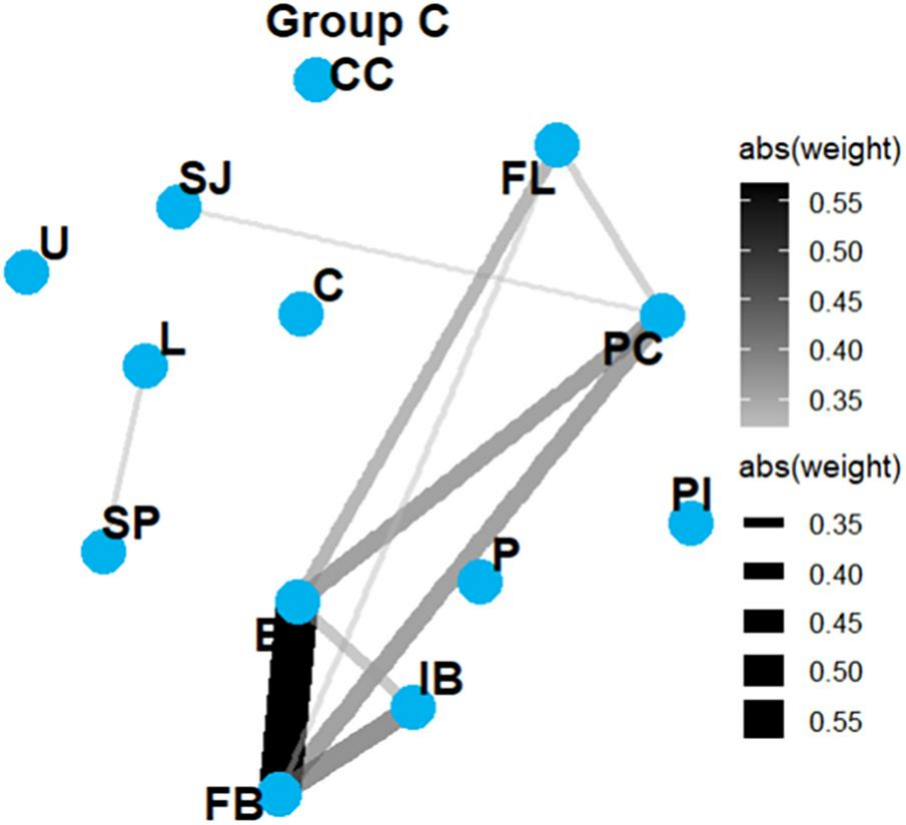
Network graphs in group C. Similarity between the AD and control networks was markedly lower (Jaccard index: 0.222; weighted Jaccard: 0.187; correlation similarity: r = 0.261), indicating distinct network organization associated with the disease state.

Network Graph Comparison: Group AD vs. Group C (Figures 6, 7).

Those graphs revealed two strongly interconnected clusters. The first cluster comprises FB, IB, P, and E, whereas the second group includes SP, L, C, U, and SJ. In contrast, FL, PI, and PC appear as isolated nodes. The disease network became more sparsely connected, with local connectivity patterns becoming less cohesive in the disease state. In contrast, similarity between the disease and control networks was substantially lower (Jaccard index: 0.222; weighted Jaccard: 0.187; correlation matrix similarity: r = 0.261), indicating distinct structural patterns.

### ML-Based classification of AD and C

#### Feature selection and model performance

The cross-validation plots for the LASSO regression and regularization paths are shown in Supplementary Figure S2. The multivariate logistic regression analysis, based on the variables selected using the LASSO technique, is presented in Table 2. B, P, PC, E, SJ, FB, and FL were positively associated with AD, whereas C and CC were negatively associated. LASSO regression with 10-fold cross-validation identified key features at *λ* = 0.0195 (*λ*.1se). Using these selected features, the Random Forest model demonstrated excellent performance under 10-fold cross-validation, with an area under the curve of 0.989, accuracy of 0.957, sensitivity of 0.931, specificity of 0.980, and a Cohen's *κ* of 0.913. The Variance Inflation Factor (VIF) values for all predictors were as follows: P, 2.00; C, 1.16; PC, 1.45; SJ, 1.57; CC, 1.17; E, 2.79; FB, 2.01; U, 1.20; FL, 1.36; SP, 1.50.

**Table 2:** Comparison of LASSO and generalized linear model regression results.

Variable	Estimate	CI_Low	CI_Upper	Estimate Std.Error	Statistic	P.Value
(Intercept)	−3.224611339	−3.73449292	−2.75895791	0.248365113	−12.9833506	1.52E-38
P	0.287112605	0.179910482	0.405088427	0.057318901	5.009038901	5.47E-07
C	-0.506365259	-0.907318518	-0.162995993	0.190867979	-2.652960756	0.007978915
PC	0.095730418	0.037489202	0.15653439	0.030296102	3.159826188	0.001578633
SJ	0.652170971	0.284948094	1.039077222	0.191844617	3.39947495	0.000675154
CC	-0.641920597	-1.050447856	-0.270061988	0.198647485	-3.231455942	0.001231613
E	0.08753608	0.024017666	0.151666055	0.032509001	2.692672055	0.007088195
FB	0.170420784	0.050967811	0.292628931	0.061518234	2.770248293	0.005601358
U	-0.224729898	-0.724417818	0.254252954	0.246432245	-0.911933816	0.361803559
FL	0.355895549	0.131366342	0.585151504	0.115496416	3.081442362	0.002060004
SP	0.147350612	-0.180063364	0.525121547	0.179260203	0.821992885	0.411080947
IB	0.732024954	0.521032587	0.956900468	0.110965961	6.596842373	4.20E-11
L	-0.167237363	-0.491554974	0.147415964	0.161817201	-1.033495589	0.301372034

#### SHAP analysis for feature importance

**Figure 8:**
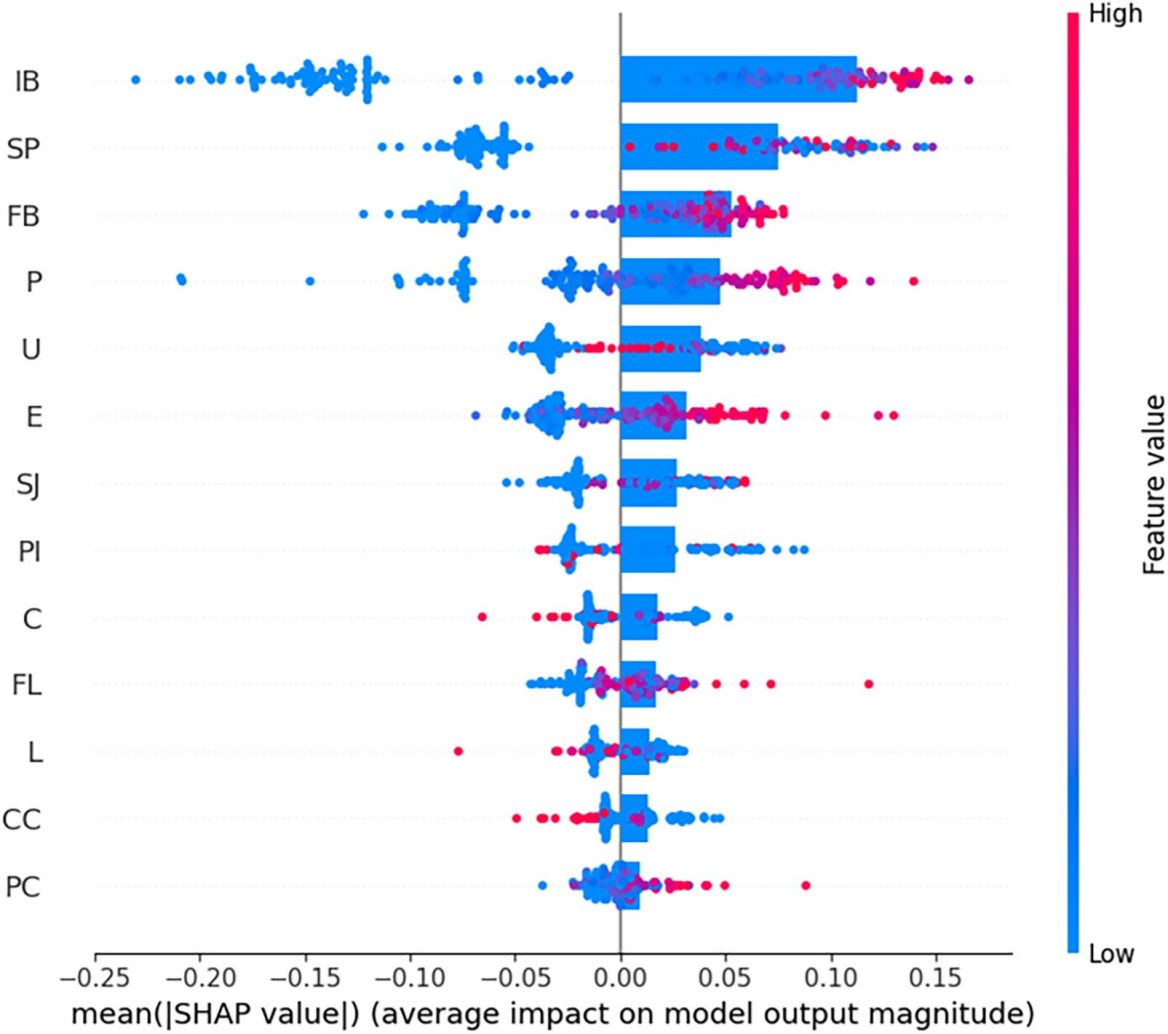
SHAP contribution analysis for random forest model positive and negative values indicate the direction and magnitude of influence on the model's output. Intramural blood (IB) had the highest impact on model predictions, followed by Salmon-pink appearance (SP), fissure bleeding (FB), puff sign **(P)**, angioscopic ulcer **(U)**, erosion **(E)**, and strawberry-jam appearance (SJ).

SHAP analysis revealed that IB, SP, and FB were the most influential features in predicting Group AD. Higher IB values contributed positively to the classification of Group AD, whereas lower FB values were associated with Group C (Figure 8).

#### Feature importance analysis using permutation importance

**Figure 9:**
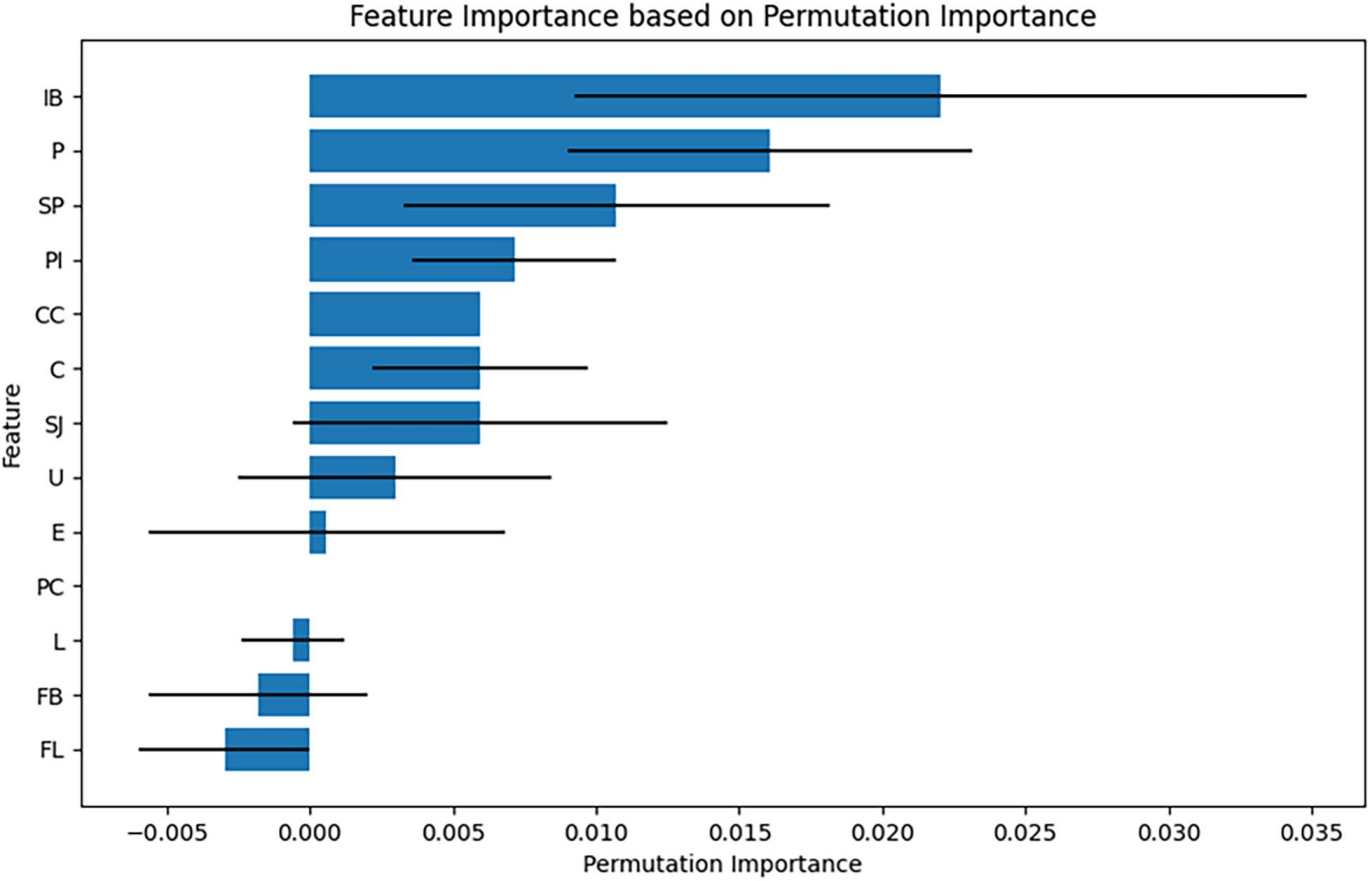
Feature importance based on permutation importance intramural blood (IB) was the most influential feature, followed by puff sign **(P)**, Salmon-pink appearance (SP), peeled intima (PI), cotton-candy appearance (CC), chandelier sign **(C)**, and strawberry-jam appearance (SJ). SRAPIs, Spontaneously ruptured aortic plaques and injuries; AD, Aortic dissection; SHAP, SHapley Additive eXplanations.

Permutation Importance analysis identified IB, P, and SP as the most influential features (Figure 9). PI, CC, C, and SJ exhibited moderate influence, contributing to the model's predictions but to a lesser extent than the top three features. In contrast, U, E, PC, L, FB, and FL had minimal impact on the model accuracy, suggesting that their removal did not significantly affect performance.

## Discussion

This study demonstrated that NOGA allows direct visualization of SRAPIs and their clustering using an ML-based approach for assessing AD. To our knowledge, this is the first study to apply network-based analysis to SRAPI relationships in the context of AD. Machine learning is often used to predict outcomes. However, it is less commonly used to explore hidden relationships between unknown factors (31). Machine learning is a useful hypothesis-generating approach for investigating findings with unclear pathological significance and phenomena that cannot be directly validated *in vivo* through biopsy (32). Among the SRAPIs, IB, P, and SP were identified as essential features strongly associated with AD. In addition, FB and E frequently coexisted in patients with AD, as confirmed by using LASSO, SHAP, and Permutation Importance analyses. Network graph analysis further revealed strong interconnections among these features. IB, P, and SP were the most significant indicators, highlighting their potential role in the early detection of AD.

IB represents subintimal hemorrhage or subintimal blood flow (10, 11, 33). SRAPIs such as IB and E are detectable only through NOGA. Initially, IB was observed at the edges of coronary artery stents after implantation (33), where it was considered an intramural hemorrhage caused by subintimal injury due to stent placement. Later, IB was also identified in the aorta of patients with AD. Notably, IB has been observed in patients before the onset of AD, despite no abnormalities being detected on CT (10). CT angiography is unable to capture FB or IB because it relies on contrast medium infiltration. Importantly, a low-density area in the aorta on CT angiography does not necessarily indicate a thrombus (34). This suggests that IB may be a previously unrecognized imaging marker that may precede the clinical onset of AD. Histopathological analysis of SRAPIs *in situ* is largely limited to PC and P (7). The vascular intima may appear frayed, and fibrin deposition may appear as *P* (12). The subintimal color of SP remains unchanged and unmovable, suggesting that SP may reflect relatively old thrombi or other subintimal components.

Some FBs function as tears, serving as entry or reentry points, whereas others separate in a membranous manner (11, 12, 14). FB was frequently observed and had the highest prevalence among SRAPIs in Group AD. However, it was not ranked as a key feature in LASSO regression, SHAP, or Permutation Importance analyses. FB was the most frequent SRAPIs in AD. A small number of FBs may contribute to AD development. As previously reported, detecting and covering FBs may be essential for reducing the pseudolumen using stent graft implantation before rupture occurs (17). In addition, bidirectional blood flow between the aortic lumen and the subintimal layer may be observed through the fissure (14). However, tears may not be visible if blood flow communication is obstructed. Therefore, continuous observation at the exact location is necessary to identify FBs based on the linear silhouette of blood flow. One possible explanation for the discordance between SHAP and permutation importance is the distinct biological nature of FB. Unlike other SRAPIs, which may reflect cumulative vascular injury, FB represents a structural fissure of the vessel wall.

It is possible that once a certain threshold of fissure formation is reached, the risk of rupture increases, potentially leading to fatal outcomes. As a result, patients with extensive FB may be underrepresented in the observed cohort, introducing a form of survivor bias.

Therefore, the number of FBs may not serve as a linear indicator of disease severity. Instead, the presence of FB may reflect a critical pathological state, whereas further increases in FB do not necessarily provide additional discriminative information.

These findings suggest that angioscopic features may persist over the observed interval rather than resolve early. However, the study was not designed or powered for formal phase comparisons, and prospective validation is required. To address class imbalance, SMOTE was used, and cross-validation confirmed a low risk of overfitting. Network graphs were compared before and after SMOTE with added noise, demonstrating that parameter relationships remained unchanged. LASSO multivariate analysis with 10-fold cross-validation effectively minimized feature loss, and validation using a random forest model confirmed the robustness of our findings. In addition, SHAP analysis and Permutation Importance provided an interpretable framework for identifying nonlinear relationships between SRAPIs and AD risk, helping to mitigate the black-box nature of ML models. The consistency of results across both linear and nonlinear statistical methods further validated the appropriateness of our variable selection. These findings confirm that SMOTE preserves the underlying network structure, while substantial differences remain between disease and control networks.

In the exploratory pre-dissection cohort, IB and E were observed in all cases, whereas P and FB were highly present. These observations are consistent with the statistical analyses that highlighted IB, E, P, and FB as key predictors of AD. Importantly, FB was not detected in every case, which may reflect that multiple or larger fissures often lead to fatal rupture and are therefore rarely observed in patients who survive to the pre-onset phase. Therefore, these SRAPIs may represent substrates that predispose to clinical aortic dissection.

Group AD showed higher SRAPIs prevalence in both qualitative and quantitative assessments than Group C. Notably, SRAPIs extended beyond CT-identified dissection sites. Future studies should explore whether a wider SRAPI distribution could serve as a potential indicator for AD recurrence or rupture risk. FB and IB were detected in the aorta before AD rupture (12), suggesting their potential role in reflecting intramural instability or persistent endothelial dysfunction. If these findings are confirmed, they could contribute to risk stratification for AD, particularly in patients with severe atherosclerosis. To validate the clinical utility of SRAPIs in predicting AD outcomes, large-scale multicenter studies are necessary.

Accordingly, the present analysis should be interpreted as hypothesis-generating rather than predictive. The limited sample size restricts the robustness of our analysis of the 13 SRAPIs. Consequently, these preliminary findings require validation in larger, multicenter studies. Case collection was maximized within practical constraints; however, further data accumulation is necessary to improve reliability. Moreover, since SMOTE generates synthetic samples rather than actual patient data, the model may not fully capture real-world conditions, underscoring the need for large-scale validation.

## Conclusions

ML models with multiple validation approaches may improve feature selection and improve the pathophysiological understanding of NOGA-based assessments for AD. Findings reflect a selected, stable-phase cohort imaged after symptom relief and blood pressure stabilization. Further multicenter validation studies are essential to confirm the generalizability of these findings.

## Data Availability

The raw data supporting the conclusions of this article will be made available by the authors, without undue reservation.

## Supplementary material

Supplementary video S1 Representative angioscopic video of SRAPIs in aortic dissection SRAPIs: Spontaneously ruptured aortic plaques and injuries
