# Supplementary material for: Exploratory machine learning analysis to characterize angioscopic features associated with atherosclerosis-related aortic dissection: an exploratory single-center angioscopic study
Source: Front Cardiovasc Med. 2026 May 7;13:1784239. doi: 10.3389/fcvm.2026.1784239 (PMC13189817; doi:10.3389/fcvm.2026.1784239)
Supplement: Supplementary file 2 [file Table1.docx]

## **Supplementary Table 1 　The definition of spontaneously ruptured aortic plaques and injuries**

| Spontaneously ruptured aortic plaques and injuries | Abbreviation | Angioscopic definition |
| --- | --- | --- |
| Puff sign | P | White or white-yellow, puff-like materials that are easily expelled spontaneously, composed of fibrin; observed in cases of plaque rupture or fibrin adhesion to the vascular surface. |
| Chandelier sign | C | Materials that glisten under the light from the tip of the fiber catheter during non-obstructive general angioscopy, wriggling but not expelled, primarily considered to represent pre-rupture plaque disruption. |
| Puff-chandelier rupture | PC | A mixture of puff rupture and chandelier appearance, composed of cholesterol crystals, atheroma, and other components. PC is observed in plaque rupture. |
| Strawberry-jam appearance | SJ | Red thrombi adhering to the surface of the aorta, resembling strawberry jam; not ulcerative and not washed out by aortic blood flow or infusion of low molecular dextran. |
| Cotton-candy appearance | CC | Solid white or red materials resembling cotton candy but immobile (Figure 2E, an arrow); calcification is a potential candidates, though challenging to confirm. |
| Angioscopic erosion | E | Erosive lesion beneath the media of the aortic wall, not necessarily identical meaning to erosion in the coronary artery |
| Fissure bleeding | FB | Elongated cleft or tear, occasionally allowing blood communication with the vessel wall; it may represent an entry or re-entry (Figure 3G, an arrow) and can remain a localized, minute blood communication in some situations. |
| Angioscopic ulcer | U | An angioscopically observed ulceration penetrating the internal elastic lamina and the media of the aortic wall. Angioscopic ulcer represents a distinct concept from penetrating atherosclerotic ulcer though deeper than erosion |
| Flap | FL | A piece of tissue, partially detached, which waves or flutters (Figure 2I, a contour) |
| Peeled intima | PI | Intima peeled in a small area (Figure 2J, an arrow) |
| Salmon-pink appearance | SP | The pink and red inner surface that is never washed out by aortic blood or infusion of low molecular dextran |
| Intramural blood | IB | Subintimal bleeding never washed out by aortic blood or infusion of low molecular dextran |
| Loft appearance | L | A cavity formed by the loss of vascular wall structure or plaque contents (Figure 2M, arrows) |
