# Supplementary material for: Exploratory machine learning analysis to characterize angioscopic features associated with atherosclerosis-related aortic dissection: an exploratory single-center angioscopic study
Source: Front Cardiovasc Med. 2026 May 7;13:1784239. doi: 10.3389/fcvm.2026.1784239 (PMC13189817; doi:10.3389/fcvm.2026.1784239)
Supplement: Supplementary file 3 [file Table2.docx]

## **Supplementary Table 2. Comparison of prevalence of SRAPIs in Groups AD and C**

SRAPIs Group AD Group C *P*_value

FB 99.5 56.1 p<0.001

E 98.2 74.1 p<0.001

P 94.9 57.4 p<0.001

PC 91.1 60.8 p<0.001

IB 90.1 21.2 p<0.001

FL 69.6 37.6 p<0.001

SP 34.4 5.6 p<0.001

SJ 30.4 14 p<0.001

L 30.1 16 p<0.001

U 29.6 10.6 p<0.001

CC 18.1 18.5 0.965

C 15.3 17.6 0.432

PI 11.2 9.7 0.539

SRAPIs, Spontaneously ruptured aortic plaques and injuries; AD, Aortic dissection; P, Puff sign; C, Chandelier sign; PC, Puff-chandelier rupture; SJ, Strawberry-jam appearance; CC, Cotton-candy appearance; E, Angioscopic erosion; FB, Fissure bleeding; U, Angioscopic ulcer; FL, Flap; PI, Peeled intima; SP, Salmon-pink appearance; IB, Intramural blood; L, Loft appearance
