# Supplementary material for: Exploratory machine learning analysis to characterize angioscopic features associated with atherosclerosis-related aortic dissection: an exploratory single-center angioscopic study
Source: Front Cardiovasc Med. 2026 May 7;13:1784239. doi: 10.3389/fcvm.2026.1784239 (PMC13189817; doi:10.3389/fcvm.2026.1784239)
Supplement: Supplementary file 4 [file Table3.docx]

# **Supplementary Table 3 Angioscopic SRAPI findings in the exploratory pre-dissection cohort**

| Case number | Gender | Age | Time to progression of aortic dissection (months) | All SRAPIs | P | C | PC | SJ | CC | E | FB | U | FL | PI | SP | IB | L |
| --- | --- | --- | --- | --- | --- | --- | --- | --- | --- | --- | --- | --- | --- | --- | --- | --- | --- |
| Case 1 | male | 83 | 48 | 39 | 0 | 1 | 7 | 0 | 1 | 12 | 8 | 0 | 0 | 0 | 5 | 4 | 0 |
| Case 2 | female | 76 | 9 | 28 | 1 | 0 | 0 | 0 | 0 | 6 | 2 | 0 | 2 | 0 | 0 | 13 | 0 |
| Case 3 | female | 75 | 24 | 29 | 3 | 1 | 2 | 1 | 0 | 10 | 0 | 0 | 3 | 0 | 0 | 2 | 0 |
| Case 4 | male | 79 | 25 | 48 | 14 | 0 | 3 | 1 | 0 | 17 | 4 | 0 | 1 | 0 | 0 | 5 | 0 |
