# Supplementary material for: Exploratory machine learning analysis to characterize angioscopic features associated with atherosclerosis-related aortic dissection: an exploratory single-center angioscopic study
Source: Front Cardiovasc Med. 2026 May 7;13:1784239. doi: 10.3389/fcvm.2026.1784239 (PMC13189817; doi:10.3389/fcvm.2026.1784239)

## **Supplementary Figure 1 Representative images of SRAPIs**

A: Puff sign (P); B: Chandelier sign (C); C: Puff-chandelier rupture (PC); D: Strawberry-jam appearance (SJ); E: Cotton-candy appearance (CC); F: Angioscopic erosion; G: Fissure bleeding (FB); H: Angioscopic ulcer (U); I: Continuous image set of the flap (FL); J: Peeled intima (PI); K: Salmon-pink appearance (SP); L: Continuous image set of intramural blood (IB); and M: Loft appearance (L). SRAPIs, Spontaneously ruptured aortic plaques and injuries


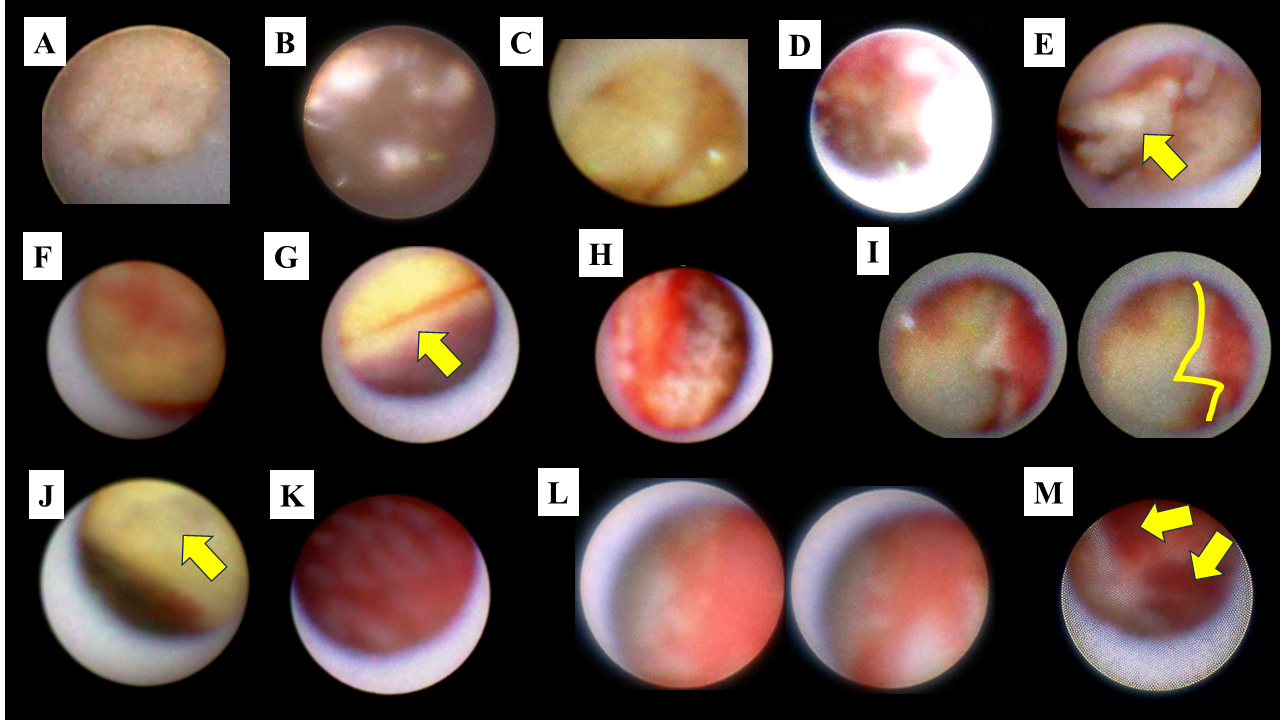

Supplement: Supplementary file 5 [file Datasheet1.docx]
