# Supplementary material for: Exploratory machine learning analysis to characterize angioscopic features associated with atherosclerosis-related aortic dissection: an exploratory single-center angioscopic study
Source: Front Cardiovasc Med. 2026 May 7;13:1784239. doi: 10.3389/fcvm.2026.1784239 (PMC13189817; doi:10.3389/fcvm.2026.1784239)
Supplement: Supplementary file 6 [file Datasheet2.docx]

## **Supplementary Figure 2. Selection and regularization path in LASSO regression for SRAPIs**

1. Cross-validation plot for LASSO Regression demonstrating the selection process for the optimal penalty coefficient (λ)
2. The LASSO regularization path for SRAPIs
3.
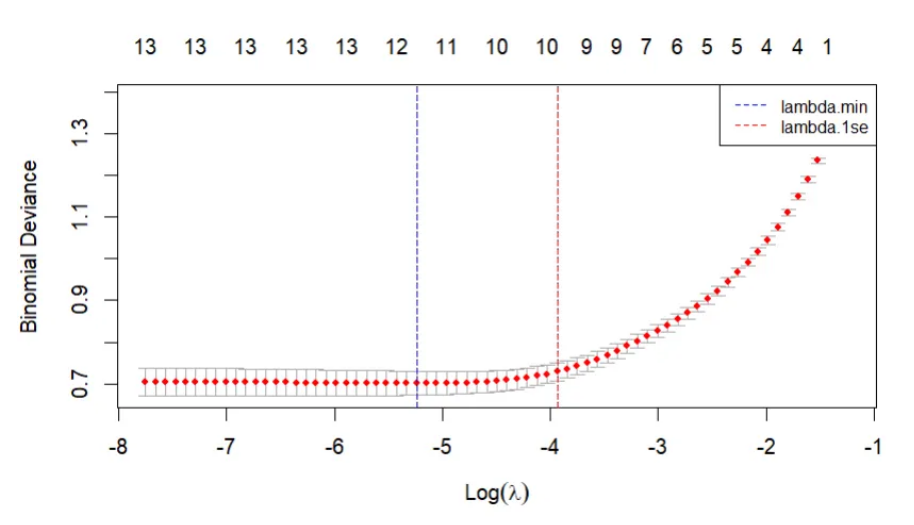


**
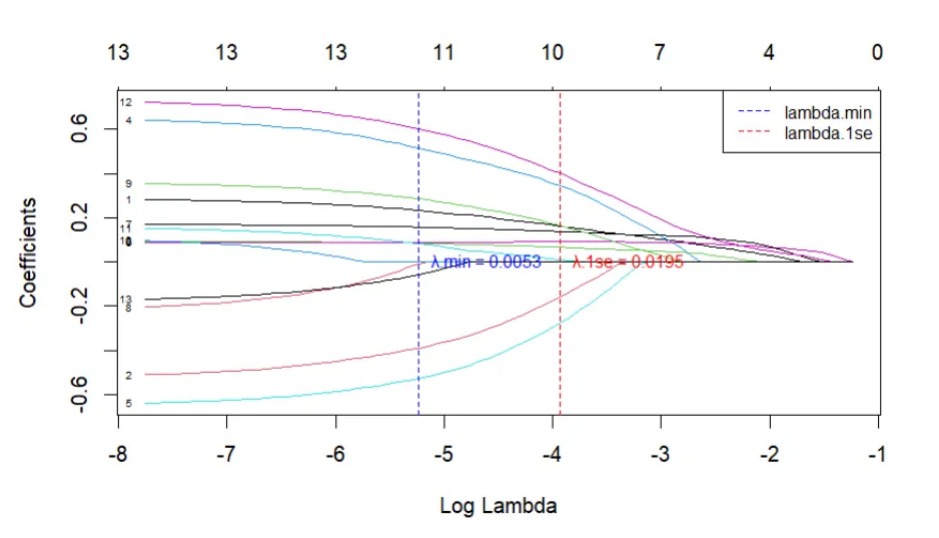
**

LASSO, Least Absolute Shrinkage and Selection Operator; SRAPIs, Spontaneously ruptured aortic plaques and injuries
